# Dr. Matthew F. Haney

**Published:** 1904-04

**Authors:** 


Dr. Matthew F. Haney, of Humberstone, Ont., University of Buffalo, 1850, died December 3, 1903, aged 79 years.

